# The Nexus of Atrial Fibrillation, Heart Failure, and Cardiac Pacing Therapies in 2022

**DOI:** 10.19102/icrm.2023.14019

**Published:** 2023-01-15

**Authors:** Imran Niazi

**Affiliations:** ^1^Aurora Cardiovascular and Thoracic Services, Aurora Sinai/Aurora St. Luke’s Medical Centers, University of Wisconsin School of Medicine and Public Health, University of Wisconsin–Madison, Madison, WI, USA

**Keywords:** Atrial fibrillation, cardiac resynchronization therapy, heart failure

Our understanding of the various forms of conduction system pacing, cardiac resynchronization therapy (CRT), and their clinical role in the treatment of heart failure continues to evolve. The previous year regaled us with a flurry of studies, which have advanced our understanding and piqued our interest in their clinical role.

The International LBBAP Collaborative Study Group reported their experience with left bundle branch pacing combined with CRT^1^ in patients in sinus rhythm. This experience involved 9 centers in 3 continents. A heterogeneous population of 112 non-sequential patients underwent placement of a permanent left bundle pacing lead and a CRT lead, with clinical and echocardiographic follow-up. Implantation was successful in 91 patients (81%), a respectable real-world number, and there were no serious complications. Echocardiographic remodeling occurred in 63%, while a clinical response was documented in 76%, a commendable result considering that the patient population included some patients who had failed to respond to CRT in the past, while others had right bundle branch block. Of interest, the echocardiographic and clinical responses were significantly superior in patients with left bundle capture versus left ventricular septal pacing when combined with CRT.

The diverse patient population prevents us from drawing too many conclusions, but these intriguing results make good theoretical sense. While the site of block is proximal in most patients with cardiomyopathy and left bundle branch block, there are varying degrees of functional block or delay distally in many, which cannot be completely overcome by left bundle pacing alone; CRT can stimulate areas with delayed conduction and complement intra-ventricular synchrony, while the proximal conduction system is activated antegrade by left bundle stimulation. Also, left bundle pacing cannot always be accomplished in every patient, and left septal pacing introduces a small but physiologically significant delay in activation of the lateral wall of the LV,^2^ which can be compensated for by CRT.

Building on the conduction system pacing theme, Huang et al. compared His-bundle pacing with CRT in patients with AF and reduced ejection fraction in the ALTERNATIVE-AF trial.^3^ This randomized prospective trial employed a crossover design; 50 patients with persistent AF, left ventricular ejection fraction < 40%, narrow QRS (or right bundle branch block), and adequate rate control (mean heart rate, 93.1 ± 19 bpm) underwent atrioventricular junction ablation and implantation of His bundle, right ventricular, and coronary sinus branch pacing leads. Patients were randomized to His-bundle pacing or biventricular CRT, with crossover at 9 months. The primary endpoint was echocardiographic remodeling, and secondary endpoints included quality of life assessment and brain natriuretic peptide measurements. Thirty-eight patients completed the trial; 7 of the 12 remaining patients died and 5 were lost to follow-up.

Among the patients who completed the trial, the overall response rate at 18 months was 94.7%, an exceptional result; only 2 patients failed to show improvement. The left ventricular ejection fraction improved from 32.6% ± 6.4% to 53.9% ± 11.9% after the first 9 months with His-bundle pacing and from 34.6% ± 4.6% to 51.3% ± 7.4% with CRT during the same period. This slightly greater response (4.5%) achieved with His-bundle pacing was statistically significant, and this difference was maintained after 18 months. The left ventricular end-diastolic dimension improved equally with both pacing modalities, as did the New York Heart Association functional class, quality of life measures, and brain natriuretic peptide levels.

This trial is remarkable in many respects. The 100% success with His and CRT leads is a testament to the skill, experience, and persistence of the implanting physician. A recent meta-analysis of His-bundle pacing trials showed a more modest 79.8% success rate.^4^ The relatively young age (mean age, 64 years) and healthy state (valve disease and severe renal dysfunction were exclusionary) of the study population support implant success, as does the absence of conduction system disease. The overall response rate of 95% also excludes the 12 patients who died or were lost to follow-up. Further, cardiomyopathy was arrhythmia-mediated in the majority, with only 2 patients having an ischemic etiology; a superior response rate is not unexpected in this population. These excellent results may not be reproducible in an older, sicker population or with other, less-experienced operators in the real world of clinical practice.

Both CRT and His pacing following atrioventricular node ablation produced excellent reverse remodeling. The slightly better response to His pacing is not unexpected since no form of biventricular CRT can replicate the perfectly synchronous contraction engendered by activating the normal conduction system. In the medium term, there was no clinical difference between His pacing and CRT in New York Heart Association class or quality of life measures. It is uncertain whether this difference will prove to be clinically important in the long term. Our experience with CRT tells us that dyssynchrony-mediated ventricular remodeling can take many years to develop. While His pacing thresholds were exceptional (0.9 ± 0.6 mA at 18 months) in the study, this has not been the general experience in other centers, where His pacing thresholds are generally significantly higher and tend to rise with time.^5^ Whether this becomes a concern over the long term remains to be seen. Longer-term follow-up during larger randomized trials will be needed to answer these questions.

Another important aspect of this trial was that the patients included did not have uncontrolled ventricular rates during atrial fibrillation (AF); the arrhythmia-induced cardiomyopathy was not necessarily rate-related but instead due to the irregularity of the ventricular rate during AF. Rate regularization rather than rate control was the likely mechanism for improvement. In this regard, Huang et al.^3^ echoed the work of Brignole and colleagues,^6,7^ who elegantly demonstrated the importance of rate regularization in the noteworthy APAF-CRT trials.

We are all aware of the negative effects of tachycardia on ventricular function, but the deleterious effects of an irregularly irregular rate are less well recognized even though the underlying physiology is well understood.^8,9^ Even strict control of the rate during AF does not ameliorate the underlying pathology, as shown in the RACE-II trial.^10^ Perfect rate regularity can be achieved by ablation of the atrioventricular node and pacing of the ventricles. In older pace-ablate trials, the benefit of rate regularity was negated by the adverse effects of right ventricular pacing–induced dyssynchrony.^11^ In the ALTERNATIVE AF and APAF-CRT trials, His-bundle pacing or CRT eliminated the adverse effects of right ventricular pacing and allowed the benefits of rate regularity to emerge. ALTERNATIVE AF showed profound reverse remodeling and symptomatic improvement, and the larger APAF-CRT trial demonstrated a striking reduction in total mortality and hospitalizations for heart failure.

These trials re-awaken our interest in adopting a pace-ablate strategy for patients with refractory AF and heart failure. Restoration of sinus rhythm with AF ablation was shown to improve survival in the landmark Catheter Ablation vs. Standard Conventional Treatment in Patients with LV Dysfunction and AF (CASTLE-AF) trial,^12^ but it must be recognized that many patients fail AF ablation. A meta-analysis evaluating the long-term outcomes of 6,167 patients who underwent a single radiofrequency ablation procedure for AF revealed that only 54.1% of paroxysmal AF patients and 41.8% of non-paroxysmal AF patients maintained sinus rhythm at 1 year.^13^ When maturation of technology allows speedy and reliable conduction system pacing, this, possibly coupled with CRT, may turn out to be the treatment of choice for such patients.
